# Qingchang Wenzhong Decoction Ameliorates Dextran Sulphate Sodium-Induced Ulcerative Colitis in Rats by Downregulating the IP10/CXCR3 Axis-Mediated Inflammatory Response

**DOI:** 10.1155/2016/4312538

**Published:** 2016-06-16

**Authors:** Tang-you Mao, Rui Shi, Wei-han Zhao, Yi Guo, Kang-li Gao, Chen Chen, Tian-hong Xie, Jun-xiang Li

**Affiliations:** ^1^Beijing University of Chinese Medicine, No. 11, North Third Ring East Road, Beijing 100029, China; ^2^Gastroenterology Department, Dongfang Hospital, Beijing University of Chinese Medicine, No. 6, 1st Section, Fangxingyuan, Fangzhuang, Beijing 100078, China

## Abstract

Qingchang Wenzhong Decoction (QCWZD) is an effective traditional Chinese medicine prescription. Our previous studies have shown that QCWZD has significant efficacy in patients with mild-to-moderate ulcerative colitis (UC) and in colonic mucosa repair in UC rat models. However, the exact underlying mechanism remains unknown. Thus, this study was conducted to determine QCWZD's efficacy and mechanism in dextran sulphate sodium- (DSS-) induced UC rat models, which were established by 7-day administration of 4.5% DSS solution. QCWZD was administered daily for 7 days, after which the rats were euthanized. Disease activity index (DAI), histological score (HS), and myeloperoxidase (MPO) level were determined to evaluate UC severity. Serum interferon gamma-induced protein 10 (IP10) levels were determined using ELISA kits. Western blotting and real-time polymerase chain reaction were, respectively, used to determine colonic protein and gene expression of IP10, chemokine (cys-x-cys motif) receptor (CXCR)3, and nuclear factor- (NF-) *κ*B p65. Intragastric QCWZD administration ameliorated DSS-induced UC, as evidenced by decreased DAI, HS, and MPO levels. Furthermore, QCWZD decreased the protein and gene expression of IP10, CXCR3, and NF-*κ*B p65. Overall, these results suggest that QCWZD ameliorates DSS-induced UC in rats by downregulating the IP10/CXCR3 axis-mediated inflammatory response and may be a novel UC therapy.

## 1. Introduction

Ulcerative colitis (UC), a major phenotype of inflammatory bowel diseases (IBD), is characterized by chronic nonspecific inflammation of the colorectal mucosa and a relapsing-remitting course [1, 2]. The number of patients with UC in Asia is increasing annually, and the incidence of UC has increased more than 3 times in the last 10 years in China [3]. Although the exact pathogenesis of UC remains unclear, it is well known that intestinal mucosal immune system disorders, intestinal mucosal barrier defects, persistent intestinal infections, and genetic and environmental factors are involved in the development of UC [4, 5]. Persistent intestinal infections in particular are a major cause of UC [6], and alleviation of infection has become the main approach in the treatment of UC.

Interferon gamma- (IFN-*γ*-) induced protein 10 (IP10) is an endogenous chemokine belonging to the CXC subfamily. Previous studies have demonstrated that the amounts of IP10 in the serum and colon tissue of UC patients were significantly higher than the corresponding amounts in healthy individuals [7]. Thus, inhibiting IP10 expression can effectively resolve the clinical symptoms of UC. Furthermore, blocking of IP10 expression was shown to alleviate spontaneous colitis induced by IL-10 knockout in mice [8, 9]. IP10 expression was shown to increase in multiple cells such as monocytes, natural killer (NK) cells, T helper (Th)1 cells, and endothelial cells (ECs) under stimulation by intestinal IFN-*γ* and tumour necrosis factor- (TNF-) *α* [10].

Chemokine (cys-x-cys motif) receptor 3 (CXCR3) is the specific binding protein of IP10, and it is mainly expressed by epithelial and endothelial cells, as well as lymphoid cells such as memory T cells, NK cells, B cells, neutrophils, and monocytes. Upon binding to IP10, CXCR3 is activated, which increases the chemotactic activity of CXCR3-positive cells and contributes to its transfer to local inflammatory lesions [11]. Finally, the intestinal mucosa produces large amounts of inflammatory cytokines [12, 13], which not only further stimulate IP10, thus recruiting more immune cells and forming a vicious cycle, but also directly or indirectly damage the intestinal mucosal barrier and the intestinal mucosa [14, 15] and promote the occurrence and development of UC [16]. Therefore, the IP10/CXCR3-mediated inflammatory response plays an important role in the pathogenesis of UC, and inhibiting the IP10/CXCR3 axis to block excessive inflammation may be a new approach for treatment of UC.

At present, an effective treatment for UC is lacking. Amino salicylic acid, steroid hormones, and immunosuppressive drugs are the main treatments for UC, and the primary goals of these treatments are to induce remission and prevent a relapse [17, 18]. However, these treatments are associated with numerous problems such as drug intolerance, side effects, requirement of a prolonged treatment course, and high recurrence rate. Thus, there is an urgent need for the development of novel and effective drugs for treatment of UC [19].

Qingchang Wenzhong Decoction (QCWZD) is a new and effective traditional Chinese medicine prescription formulated by Li Jun-Xiang, a professor at the Beijing University of Chinese Medicine. Our previous studies have shown significant clinical efficacy of QCWZD in patients with mild-to-moderate UC [20, 21]. Furthermore, in rat models of UC, QCWZD reduced damage to colonic epithelial cells, repaired the colonic mucosa, and downregulated proinflammatory cytokines [22, 23]. However, the exact mechanism of action is yet to be elucidated. Therefore, the present study sought to further explore the molecular mechanism underlying QCWZD's protective effects in rat models of UC.

## 2. Materials and Methods

### 2.1. Preparation of QCWZD and Mesalazine

QCWZD granules were purchased from the Pharmacy Department of Dongfang Hospital, Beijing University of Chinese Medicine (Beijing, China). The QCWZD granules contained Qingchang Wenzhong ingredients in equal weights: Huanglian (coptis), 6 g; Pao Jiang (ginger), 10 g; Kushen (matrine), 15 g; Qingdai (indigo), 6 g; Diyutan (sanguisorba carbon), 30 g; Muxiang (wood), 6 g; Sanqi (pseudoginseng), 6 g; and Gancao (licorice), 6 g. Mesalazine was purchased from Losan Pharma GmbH, Germany.

### 2.2. Experimental Animals

All the experimental procedures were approved by the Animal Ethics Committee of Beijing University of Chinese Medicine, in accordance with guidelines issued by Regulations of Beijing Laboratory Animal Management. Fifty male Sprague-Dawley rats (weighing 180–220 g; SPF Biological Technology Co., Ltd., Beijing, China) were housed in a specific pathogen-free animal room with temperature maintained at 20–24°C, 50–60% humidity, and a light-controlled environment (12/12 h light/dark cycle), with free access to food and sterile tap water. All animals were allowed to adapt for 7 days before the experiments were started.

### 2.3. Induction of Colitis by DSS and Experimental Procedure

Colitis was induced using 4.5% (w/v) DSS (MW, 36–50 KDa, MP Biomedical, California, USA) added to distilled water. Fresh DSS solution was administered every day. The rats in the DSS (DSS, *n* = 10), low-dose QCWZD (low, 0.3 g/kg/day, p.o., *n* = 10), high-dose QCWZD (high, 1.2 g/kg/day, p.o., *n* = 10), and mesalazine (mesalazine, 0.03 g/kg/day, p.o., *n* = 10) groups were administered 4.5% DSS solution from day 1 to day 7. The control group rats received only distilled water. All rats were killed after deep anaesthesia with 10% chlorine hydrate solution (3.5 mL/kg, i.p.), and blood samples were collected from the abdominal aorta. Colon samples (8 cm in length) were removed and cut into 6 segments, each measuring 0.5 cm, starting from the anus and cut at 1 cm intervals. One of every two sections was fixed in 10% neutral buffered formalin for histological analysis, and the other one was placed into a freezer tube and stored at −80°C until used.

### 2.4. Analysis of Disease Activity Index

All rats were checked daily for colitis by evaluating their weight, faecal occult blood, and stool consistency. The disease activity index (DAI) was evaluated daily in a blinded manner by nonproject team members (Table 1) as previously described [24]. DAI was calculated using the following formula: DAI = (percent weight loss score + stool consistency score + haematochezia level score)/3.

### 2.5. Histological Analysis

After being fixed with 10% neutral buffered formalin for 24 h, the tissues were embedded in paraffin blocks and sectioned to obtain a thickness of 6 *μ*m. Thereafter, the sections were stained with haematoxylin and eosin (HE), and the histological score (HS) was determined using an optical microscope (Table 2). Each sample was randomly selected from 3 perspectives, and the average score was calculated as previously described [25]. The analysis was performed by nonproject team members under the guidance of a pathologist.

### 2.6. Assay for Myeloperoxidase (MPO) Activity

Frozen colons that were dissected 1 cm above the anus were homogenized in phosphate-buffered saline and centrifuged at 20,000 ×g. Then, myeloperoxidase (MPO) activity in the colonic tissue was detected using chemical colorimetry (NANODROP 2000, Thermo, USA) as previously described [26].

### 2.7. Measurement of Serum IP10 Level Using ELISA

After blood samples were collected from the abdominal aorta of rats, IP10 levels in the serum were tested using rat ELISA kits (MULTISKAN MK3, Thermo, USA).

### 2.8. Western Blotting for Detection of IP10, CXCR3, and NF-*κ*B p65 Expression in the Colon

Western blot analysis was performed as described previously [27]. Proteins were isolated from ice-cold colon tissues, and protein concentrations were determined using the bicinchoninic acid (BCA) assay (Cwbiotech, Beijing, China). The proteins were then separated using 10% SDS-PAGE for 1.5 h before being transferred to polyvinylidene fluoride (PVDF) membranes. The membranes were probed with IP10 (1 : 5000), CXCR3 (1 : 5000), NF-*κ*B p65 (1 : 2000), and GAPDH (1 : 1000) antibodies (TDY Biotech, Beijing, China). Each membrane was washed three times for 10 min and incubated with goat polyclonal secondary antibody to rabbit antibodies (111-035-003, Jackson Laboratory, USA). Finally, densitometry was performed to quantitate protein band intensities by using the Gel Image System ver. 4.00 (Tanon, China).

### 2.9. Real-Time Polymerase Chain Reaction for IP10, CXCR3, and NF-*κ*B p65 mRNA Expression

Evaluation of mRNA expression was performed by using real-time polymerase chain reaction as previously described [28]. Total RNA was extracted from the colon tissue samples by using extraction kits (CWbio, Beijing, China). After reverse transcription, PCR amplification was performed using the TRIzol method (TRIzol reagent; Invitrogen Life Technologies, Carlsbad, CA, USA). The real-time PCR primer sequences for target genes were as follows: 5′-GCGGCTAGTCCTAACTGTCC-3′/5′-GAATTGGGAAGCCTTGCTGC-3′ for IP10, 5′-TCACTTCCTCTGTTCACGGC-3′/5′-AGGAGGCTGTAGAGGACTGG-3′ for CXCR3, 5′-CAGACACCTTTGCACTTGGC-3′/5′-CTTGAGTAGGACCCCGAGGA-3′ for NF-*κ*B p65, and 5′-CCCATCTATGAGGGTTACGC-3′/5′-TTTAATGTCACGCACGATTTC-3′ for GAPDH (CWbio, Beijing, China). For real-time PCR, the cycling conditions were 95°C for 10 min and 45 × (95°C for 10 s, 59°C for 60 s), followed by a melting curve analysis-based assay with conditions of 95°C for 15 s and 72°C for 15 s and increase in temperature to 95°C for 15 s. Relative expression was assessed by calculating the expression relative to that of GAPDH by using the 2^−ΔΔCt^ method.

### 2.10. Statistical Analysis

All data are expressed as mean ± standard deviation (SD) values. SPSS v18.0 (IBM Corp., Armonk, NY, USA) was used for statistical analyses. The data were compared between groups using one-way analysis of variance (ANOVA), followed by Student's *t*-tests. *P* < 0.05 was considered statistically significant.

## 3. Results

### 3.1. Effect of QCWZD on Body Weight and DAI in UC Rats

**Figure 1:**
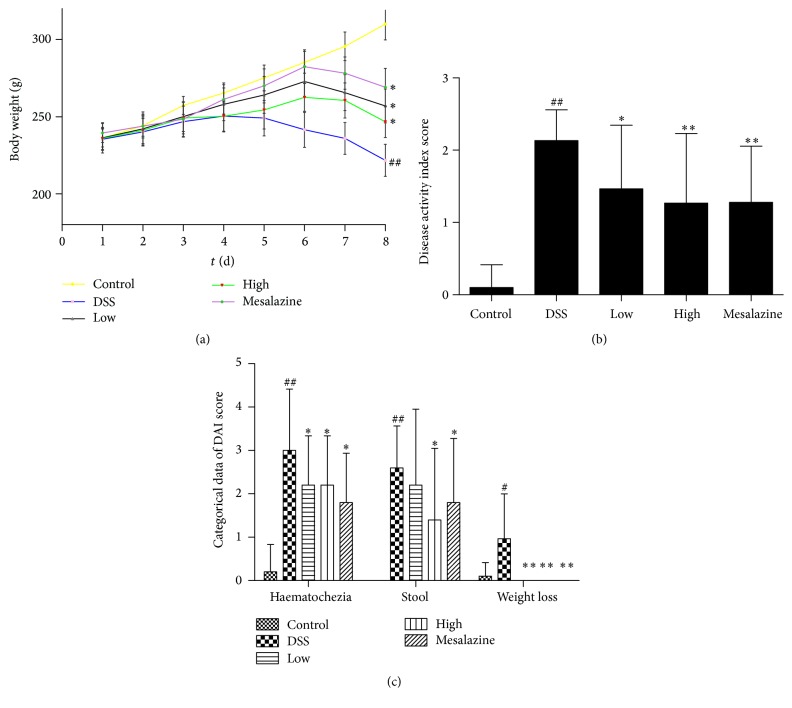
Effect of QCWZD on body weight (a), DAI score (b), and categorical data of DAI (c) in UC rats. Control: blank control group; DSS: DSS-treated group; low: low-dose QCWZD group; high: high-dose QCWZD group; mesalazine: mesalazine group; haematochezia: haematochezia level; stool: stool consistency; and weight: percent weight loss. Data are presented as the mean ± SD. ^##^ *P* < 0.01, ^#^ *P* < 0.05 versus the control group; ^*∗∗*^ *P* < 0.01, ^*∗*^ *P* < 0.05 versus the DSS group (*n* = 10 per group).

All animals tolerated the entire experiment well, and no deaths occurred. After free access to 4.5% DSS for 4 days, body weight started to decrease in the DSS group. By the 8th day, the weight loss was significantly higher than that in the control group (DSS group versus control group: 221.70 ± 10.35 g versus 310.00 ± 10.21 g, *P* < 0.01). Both the low-dose (256.90 ± 10.96 g) and high-dose QCWZD (246.70 ± 10.140 g) groups showed significant attenuation of body weight loss (*P* < 0.05, resp.), similar to the mesalazine group (269.10 ± 12.260 g versus 221.70 ± 10.35 g, *P* < 0.05) (Figure 1(a)). There were no significant differences between low-dose and high-dose QCWZD (*P* > 0.05), possibly because of the small sample size.

On the 8th day, the DSS group showed a significantly increased DAI score (2.13 ± 0.42) compared to that of the control group (0.10 ± 0.32; *P* < 0.01, Figure 1(b)). The DAI scores in the low-dose group (1.47 ± 0.88), high-dose group (1.27 ± 0.96), and mesalazine group (1.28 ± 0.78) were significantly lower than that in the DSS group (*P* < 0.05, *P* < 0.01, resp., Figure 1(b)).

Subsequently, we separately analysed the categorical data of DAI according to each category. As shown in Figure 1(c), the haematochezia level (3.00 ± 1.41), stool consistency (2.60 ± 0.97), and percent weight loss (0.80 ± 1.03) in the DSS group were significantly higher than those in the control group (0.20 ± 0.63, 0.00 ± 0.00, and 0.10 ± 0.32, *P* < 0.05 and *P* < 0.01, resp., Figure 1(c)). The haematochezia level in the low-dose group (2.20 ± 1.16), high-dose group (2.20 ± 1.16), and mesalazine group (1.80 ± 1.14) was significantly lower than that in the DSS group (*P* < 0.05, *P* < 0.01, resp., Figure 1(c)). The stool consistency (2.20 ± 1.75 in the low-dose group, 1.40 ± 1.65 in the high-dose group, and 1.80 ± 1.48 in the mesalazine group) and percent weight loss (0.00 ± 0.00 in the low-dose, high-dose, and mesalazine groups) showed the same tendency (*P* < 0.05 and *P* < 0.01, resp., Figure 1(c)).

### 3.2. QCWZD Improved Histopathology in Rats

**Figure 2:**
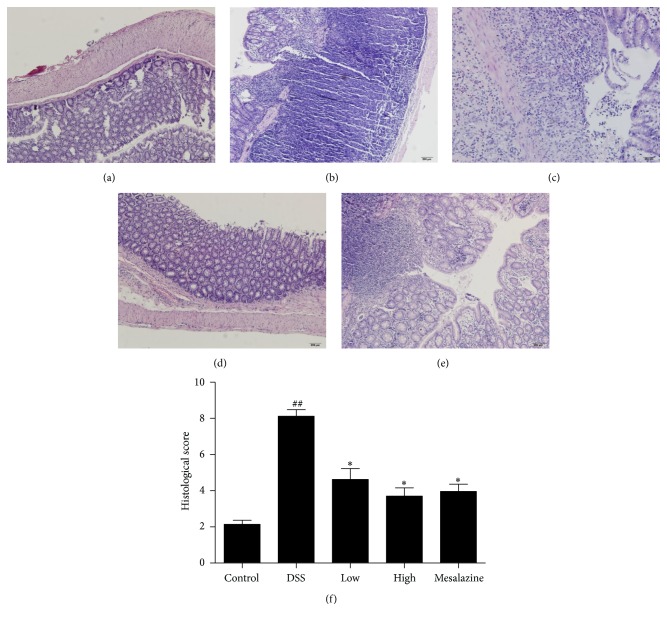
QCWZD ameliorated histological damage (a–e) and reduced the histological score (f) in rats. (a) Control: blank control group; (b) DSS: DSS group; (c) low: low-dose QCWZD group; (d) high: high-dose QCWZD group; and (e) mesalazine: mesalazine group. ^##^ *P* < 0.01 versus the control group; ^*∗*^ *P* < 0.05 versus the DSS group (*n* = 10 per group).

Compared with the control group (Figure 2(a)), the DSS group showed severely damaged crypts and epithelial integrity, and remarkable inflammatory cell infiltration in the mucosa (Figure 2(b)). After intrarectal administration of QCWZD and mesalazine, mild infiltration of inflammatory cells, crypt regeneration, and epithelial restoration were observed (Figures 2(c), 2(d), and 2(e)). Similarly, as shown in Figure 2(f), the histological score reached a significantly higher value in the DSS group (8.13 ± 0.37) than in the control group ((2.15 ± 0.23), *P* < 0.01). In contrast to the DSS group, the low- and high-dose QCWZD groups and the mesalazine group showed protection against histological damage (4.64 ± 0.60, 3.71 ± 0.46, and 3.97 ± 0.40, resp., *P* < 0.05) (Figure 2(f)).

### 3.3. QCWZD Decreased Colonic MPO Activity in DSS-Induced UC Rats

**Figure 3:**
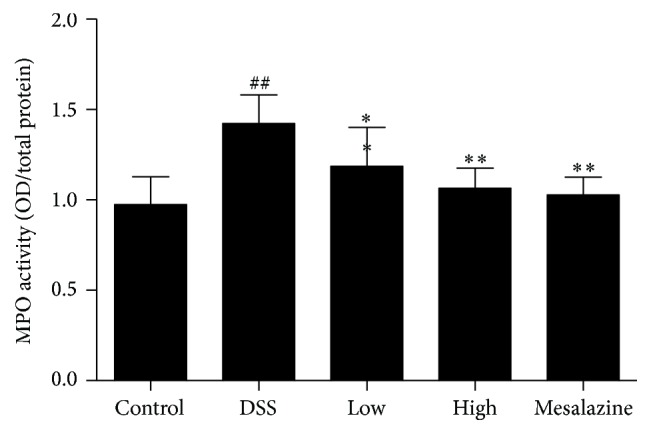
QCWZD decreased colonic MPO activity in rats with DSS-induced UC. Control: blank control group; DSS: DSS group; low: low-dose QCWZD group; high: high-dose QCWZD group; mesalazine: mesalazine group. ^##^ *P* < 0.01 versus the control group; ^*∗∗*^ *P* < 0.01, ^*∗*^ *P* < 0.05 versus the DSS group (*n* = 10 per group).

To determine the degree of infiltration by neutrophil granulocytes in the colonic tissue, the activity of MPO was detected by chemical colorimetry. Compared with the control group (0.98 ± 0.06), the DSS group showed significantly higher MPO activity (1.43 ± 0.16, *P* < 0.05). As expected, the difference was significantly less when the groups given QCWZD (1.19 ± 0.22 in the low-dose group, 1.07 ± 0.11 in the high-dose group, *P* < 0.05, *P* < 0.01, resp.) and mesalazine (1.03 ± 0.09, *P* < 0.01) were compared to the DSS group (Figure 3).

### 3.4. Effects of QCWZD on Serum IP10 Level

**Figure 4:**
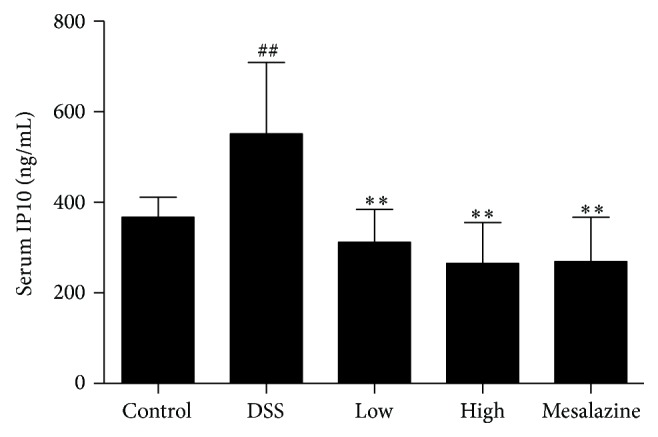
Effects of QCWZD on serum IP10 level. Control: blank control group; DSS: DSS group; low: low-dose QCWZD group; high: high-dose QCWZD group; mesalazine: mesalazine group. ^##^ *P* < 0.01 versus the control group; ^*∗∗*^ *P* < 0.05 versus DSS group (*n* = 10 per group).

The serum IP10 level was determined in the control rats and DSS-induced UC rats treated with distilled water, low- and high-dose QCWZD groups, and mesalazine group. As shown in Figure 4, DSS significantly elevated IP10 levels in serum compared with those in the control group (552.8 ± 158.5 ng/mL in the DSS group versus 367.8 ± 44.30 ng/mL, *P* < 0.01). The low-dose QCWZD group (313.0 ± 72.69 ng/mL), the high-dose QCWZD group (266.7 ± 89.99 ng/mL), and the mesalazine group (270.0 ± 98.34 ng/mL) showed significant inhibition of DSS-induced elevation of serum IP10 level (versus the DSS group, all *P* < 0.01) (Figure 4).

### 3.5. QCWZD Regulated Colonic IP10 and IP10 mRNA Expression in DSS-Induced UC Rats

**Figure 5:**
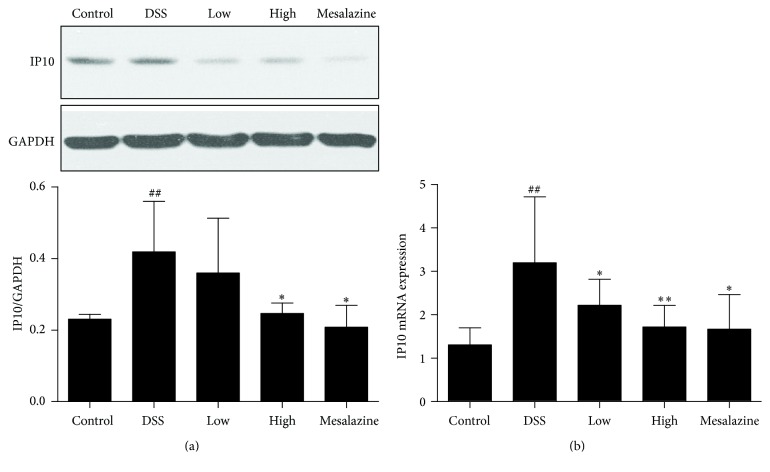
QCWZD regulated colonic IP10 (a) and IP10 mRNA (b) expression in DSS-induced UC rats. Control: blank control group; DSS: DSS group; low: low-dose QCWZD group; high: high-dose QCWZD group; and mesalazine: mesalazine group. ^##^ *P* < 0.01 versus control group; ^*∗∗*^ *P* < 0.01, ^*∗*^ *P* < 0.05 versus DSS group (*n* = 10 per group).

We also investigated whether or not QCWZD had a regulatory effect on IP10 expression in the colon. IP10 levels in the colon were significantly increased in the DSS group (*P* < 0.01 versus the control group). Additionally, high-dose QCWZD and mesalazine produced a dramatically reduced effect on IP10 expression (Figure 5(a)).

Furthermore, we measured IP10 gene expression to confirm the effects of QCWZD on the colon. As shown in Figure 5(b), induction of colitis significantly elevated colonic IP10 gene expression compared with that in the control group (*P* < 0.01). Treatment with QCWZD and mesalazine for 7 days decreased IP10 gene expression in a dose-dependent manner (*P* < 0.05, *P* < 0.01, resp.) compared to that in the DSS group.

### 3.6. QCWZD Decreased Colonic CXCR3 and CXCR3 mRNA Expression in Rats with DSS-Induced UC

**Figure 6:**
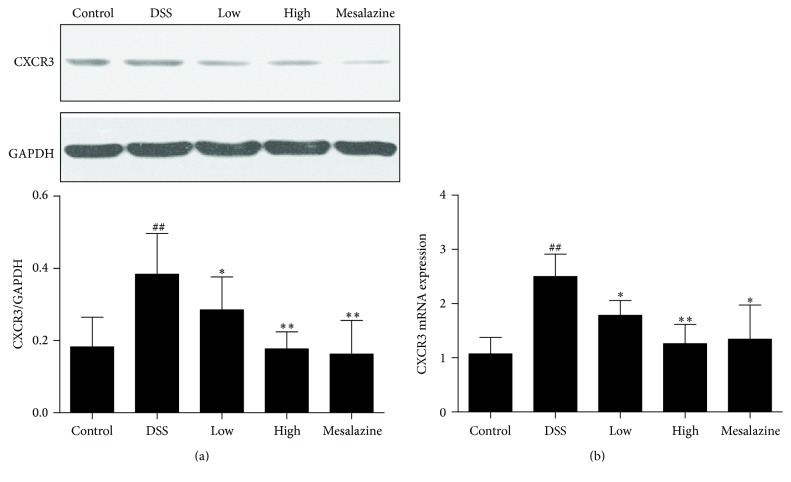
QCWZD inhibited colonic CXCR3 (a) and CXCR3 mRNA (b) expression in rats with DSS-induced UC. Control: blank control group; DSS: DSS group; low: low-dose QCWZD group; high: high-dose QCWZD group; and mesalazine: mesalazine group. ^##^ *P* < 0.01 versus control group; ^*∗∗*^ *P* < 0.01, ^*∗*^ *P* < 0.05 versus DSS group (*n* = 10 per group).

Because CXCR3 specifically binds to IP10, its levels were analysed by western blot. Compared to the control group, the DSS group showed increased CXCR3 expression (*P* < 0.01); the QCWZD and mesalazine groups showed alleviation of the effects of DSS on CXCR3 expression in rats with DSS-induced UC (versus the DSS group, *P* < 0.05 and *P* < 0.01, resp., Figure 6(a)). The expression of the CXCR3 gene was measured as described above. Gene expression in the DSS group was increased compared to that in the control group (*P* < 0.01, Figure 6(b)). Remarkably, QCWZD and mesalazine both greatly decreased CXCR3 gene expression (versus the DSS group, *P* < 0.05 and *P* < 0.01, resp., Figure 6(b)).

### 3.7. QCWZD Suppressed the Increase in Colonic NF-*κ*B p65 and NF-*κ*B p65 mRNA Expression in Rats with DSS-Induced UC

**Figure 7:**
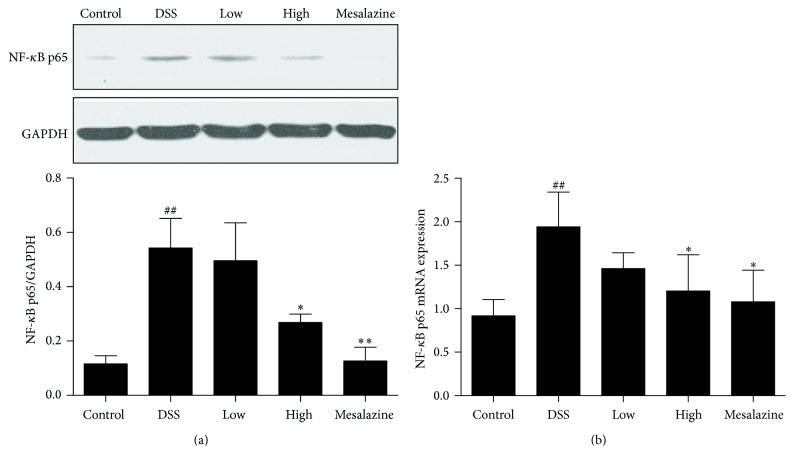
QCWZD suppressed the increase in colonic NF-*κ*B p65 (a) and NF-*κ*B p65 mRNA (b) levels in rats with DSS-induced UC. Control: blank control group; DSS: DSS group; low: low-dose QCWZD group; high: high-dose QCWZD group; and mesalazine: mesalazine group. ^##^ *P* < 0.01 versus control group; ^*∗∗*^ *P* < 0.01, ^*∗*^ *P* < 0.05 versus DSS group (*n* = 10 per group).

Because the cytokines TNF-*α* and IL-6 produced by CXCR3-positive cells contribute to the activation of NF-*κ*B, we examined the effect of CXCR3 on NF-*κ*B p65 activation. We found distinctly increased NF-*κ*B p65 and NF-*κ*B p65 mRNA expression in the DSS group compared to that in the control group (*P* < 0.01, Figures 7(a) and 7(b)). Nevertheless, NF-*κ*B p65 and NF-*κ*B p65 mRNA expression was significantly reduced in DSS-induced UC rats treated with QCWZD and mesalazine (*P* < 0.05, resp., Figures 7(a) and 7(b)).

## 4. Discussion 

In the present study, to observe the anti-inflammatory effects of QCWZD, we generated an ulcerative colitis model in rats by allowing them to drink 4.5% DSS freely for 7 days; this model is widely used because of its similarities to human UC. DSS is a polysaccharide extracted from sucrose synthesis. Although the exact mechanism by which it induces UC remains unclear, it may be related to the negative charge of DSS, which may affect the synthesis of colonic epithelial DNA, inhibit the proliferation of epithelial cells, and damage the intestinal mucosal barrier [29].

In our study, faecal occult blood began to appear on the 2nd day and the animals' body weight started to decrease on the 4th day in the DSS group. With time, gross blood began to appear in the stool and the body weight continued to decrease, and the DAI score increased and reached the maximum value on the 8th day. In the histological analysis, we found that the number of inflammatory cells (mainly neutrophils) was increased in the colonic tissue of rats that received DSS and that the crypts and epithelial integrity were severely damaged. Correspondingly, the histological score increased to a significantly higher value in the DSS group than in the control group. After administration of QCWZD and mesalazine, the body weight loss, DAI score, and histological score were significantly decreased and improved (Figures 1 and 2). These results suggest that QCWZD has prominent therapeutic effects on rats with DSS-induced ulcerative colitis.

After observing a definite effect, we further explored the therapeutic mechanism of QCWZD in rats with DSS-induced UC. Persistent intestinal infection plays an important role in the pathogenesis and development of UC [30, 31], and the IP10/CXCR3 axis that regulates the infiltration of immune cells to sites of inflammatory injury plays an essential role in the inflammatory response. Previous studies showed that both IP-10 expression in colonic tissue and IP10 levels in serum were higher in patients with UC than in healthy controls [16, 32], and that anti-IP-10 antibodies alleviated acute colitis and enhanced crypt cell survival in mice with UC induced by DSS [8]. Thus, anti-IP-10 therapy may provide an important direction for the treatment of UC. A recent phase II study for UC [7] showed that anti-IP-10 therapy with BMS-936557 effectively relieved clinical symptoms and increased histological improvement in patients with moderate-to-severe UC. Although robust efficacy was demonstrated, the drug dose response and safety profile require further research. Therefore, it is necessary to identify a new method to evaluate the therapeutic value of targeting the IP/CXCR3 axis for UC. In this regard, traditional Chinese medicine has been proven to have a curative effect in the treatment of ulcerative colitis [5, 33]. Previous studies have shown significant clinical efficacy of QCWZD in patients with UC, but the specific mechanism is not clear. Thus, we designed the present study to determine whether or not QCWZD exerts an anti-inflammatory effect through IP/CXCR3 axis. In the present study, abnormally high expression of IP10 and CXCR3 was observed in the colonic mucosa of rats with DSS-induced UC, and treatment with QCWZD and mesalazine dose-dependently decreased IP10 and CXCR3 expression (Figures 4, 5, and 6).

An increase in neutrophils is a key feature in the pathogenesis of UC [34, 35]. Coupling of IP10 to CXCR3 in the colon leads to activation of CXCR3, which promotes recruitment of neutrophils to the inflammatory site, whereas CXCR3 blockade limits neutrophil accumulation in the inflamed site. Therefore, we evaluated the expression of MPO in colon tissues, which reflects the degree of mucosal neutrophil infiltration [36, 37]. As expected, DSS significantly increased MPO activity in the colon tissue, and QCWZD treatment partially reversed this effect (Figure 3).

In addition to promoting the recruitment of neutrophils, the IP/CXCR3 axis increases the chemotactic activity of CXCR3-positive cells, which are directly and indirectly involved in the generation and recruitment of inflammatory cytokines [38] such as TNF-*α* and MCP-1, thereby damaging the intestinal mucosal barrier and colon mucosa [39, 40]. NF-*κ*B is an upstream regulator for TNF-*α* and MCP-1, which can in turn activate NF-*κ*B [41]. NF-*κ*B activation is involved in inflammatory recruitment [42]; therefore, we investigated whether or not the IP10/CXCR3 axis plays a role in NF-*κ*B activation in the development of UC. Overexpression of NF-*κ*B p65 has been identified in the colonic mucosa and is positively correlated with the increase in IP10/CXCR3. In the present study, QCWZD and mesalazine played a major role in decreasing NF-*κ*B p65 expression (Figure 7). Although other signalling pathways may also activate NF-*κ*B or proinflammatory cytokine genes, the proinflammatory effect of IP10/CXCR3 axis in UC is at least partly related to the induction of NF-*κ*B. NF-*κ*B activation is an important way that the IP10/CXCR3 axis exerts its proinflammatory effect.

In the present study, using a DSS-induced rat colonic mucosal injury as a model, we found that QCWZD significantly improved the body weight, DAI score, HS, and MPO activity in DSS-induced UC rats. The protective effects of QCWZD may be attributed to its significant regulation of the IP10/CXCR3 axis-mediated inflammatory response. QCWZD can reverse the dysregulated expression of serum and colonic IP10 and IP10 mRNA, reduce colonic CXCR3 and CXCR3 mRNA production, and thereby inhibit activation of the IP10/CXCR3 axis, in turn decreasing the generation and recruitment of inflammatory cytokines and promoting epithelial cell proliferation and repair of injured mucosa.

## 5. Conclusions

Our results show that intragastric administration of QCWZD can ameliorate DSS-induced UC in rats. The protective mechanism may be attributed to significant downregulation of the IP10/CXCR3 axis-mediated inflammatory response. Our results suggest that QCWZD may be a novel therapeutic option for UC.

## Figures and Tables

**Table 1:** DAI scoring criteria.

Types	0	1	2	3	4
Percent weight loss	0	1%–5%	5%–10%	10%–15%	>15%
Stool consistency	Normal	/	Mushy	/	Diarrhoea
Haematochezia level	Negative	/	Positive	/	Blood traces visible in stool

**Table 2:** Histological score criteria.

Histological changes	0	1	2	3
Inflammatory cell infiltration	No	Mild	Moderate	Severe
Granuloma	No	Mild	Moderate	Severe
Lesion depth	Mucosa	Submucosa	Muscular layer	Serosa
